# A Low-Cost Wearable Device to Estimate Body Temperature Based on Wrist Temperature

**DOI:** 10.3390/s24061944

**Published:** 2024-03-18

**Authors:** Marcela E. Mata-Romero, Omar A. Simental-Martínez, Héctor A. Guerrero-Osuna, Luis F. Luque-Vega, Emmanuel Lopez-Neri, Gerardo Ornelas-Vargas, Rodrigo Castañeda-Miranda, Ma. del Rosario Martínez-Blanco, Jesús Antonio Nava-Pintor, Fabián García-Vázquez

**Affiliations:** 1 Subdirección de Investigación, Centro de Enseñanza Técnica Industrial, C. Nueva Escocia 1885, Guadalajara 44638, Mexico; mmata@ceti.mx; 2 Posgrado en Ingeniería y Tecnología Aplicada, Unidad Académica de Ingeniería Eléctrica, Universidad Autónoma de Zacatecas, Zacatecas 98000, Mexico; 33145571@uaz.eud.mx (O.A.S.-M.); hectorguerreroo@uaz.edu.mx (H.A.G.-O.); ornelas@uaz.edu.mx (G.O.-V.); rcastm@uaz.edu.mx (R.C.-M.); mrosariomb@uaz.edu.mx (M.d.R.M.-B.); jesus.nava@uaz.edu.mx (J.A.N.-P.); 31126593@uaz.edu.mx (F.G.-V.); 3 Department of Technological and Industrial Processes ITESO AC, Tlaquepaque 45604, Mexico; luisluque@iteso.mx; 4 Centro de Investigación, Innovación y Desarrollo Tecnológico CIIDETEC-UVM, Universidad del Valle de México, Tlaquepaque 45601, Mexico

**Keywords:** COVID-19, machine learning, linear regression, body temperature, Internet of Things, wearable device

## Abstract

The remote monitoring of vital signs and healthcare provision has become an urgent necessity due to the impact of the COVID-19 pandemic on the world. Blood oxygen level, heart rate, and body temperature data are crucial for managing the disease and ensuring timely medical care. This study proposes a low-cost wearable device employing non-contact sensors to monitor, process, and visualize critical variables, focusing on body temperature measurement as a key health indicator. The wearable device developed offers a non-invasive and continuous method to gather wrist and forehead temperature data. However, since there is a discrepancy between wrist and actual forehead temperature, this study incorporates statistical methods and machine learning to estimate the core forehead temperature from the wrist. This research collects 2130 samples from 30 volunteers, and both the statistical least squares method and machine learning via linear regression are applied to analyze these data. It is observed that all models achieve a significant fit, but the third-degree polynomial model stands out in both approaches. It achieves an R^2^ value of 0.9769 in the statistical analysis and 0.9791 in machine learning.

## 1. Introduction

Chronic diseases are becoming a major issue worldwide, resulting in a rising need for medical treatment and services. Traditional medical practices diagnose these diseases and abnormalities in the human body. For instance, hospitals conduct physiological tests that can lead to extended hospital stays for patients during their recovery [1,2,3]. This could be observed with the most recent pandemic caused by the Coronavirus Disease 2019 (COVID-19). Recent events’ rapid spread and clinical evolution have highlighted significant deficiencies in hospital infrastructure and preparedness worldwide. As a result, there is an urgent need for more accessible and efficient medical solutions, leading to re-evaluating traditional practices. This situation has prompted the search for innovative methods that enable faster diagnosis and treatment and more effective prevention and monitoring systems, which can be implemented outside the hospital environment. This will facilitate access to medical care and improve the ability to respond to future health emergencies [4,5,6,7].

As a result of the pandemic, telemedicine and compact smart devices have been used to monitor important medical indicators and detect diseases. These devices act as remote assistants, allowing patients to be monitored outside hospitals. Integrating these technologies is a significant advancement in remote health management, providing an effective alternative for continuous patient monitoring, especially when direct access to medical services is limited [8,9,10]. Monitoring vital signs is essential for maintaining patients’ health inside and outside the hospital, and using sensors with remote communication is proposed for this purpose through technologies such as the Internet of Things (IoT) and wearable devices (WDs) [11].

IoT networks have been a popular research area for decades. This technology has evolved and adapted to optimize task allocation using various criteria such as network duration, latency, and reliability [12]. The healthcare sector has greatly benefited from the IoT revolution, as sensors, devices, and actuators allow remote patient monitoring. This technology offers a variety of applications and utilities for displaying and storing physiological data such as body temperature, heart rate, and blood oxygenation [13,14].

WD comes with sensors that can interpret physiological signals via electrical impulses. These devices find widespread usage in the healthcare sector, as well as in certain industries and sports fields [15]. In recent years, there has been a significant increase in the use of wearable biomedical sensors in healthcare, with a focus on monitoring vital signs in real time to manage chronic diseases [16,17].

Body temperature is a vital sign in evaluating an individual’s health. Changes in body temperature are instrumental in identifying major public health events, as evidenced by historical cases such as Severe Acute Respiratory Syndrome (SARS) and the recent COVID-19 pandemic [18]. Therefore, monitoring and measuring body temperature are essential to observing daily medical care, disease diagnosis, and administering advanced medical interventions.

WD has become increasingly popular for tracking various personal health metrics. One such device is designed to be worn on the wrist, providing comfort and flexibility and allowing for the continuous monitoring of vital health data with an easy and non-invasive way to collect individual information, such as body temperature [19]. It is important to note that there is a difference between the temperature measured at the wrist and the actual body temperature. According to [20], the wrist temperature’s accuracy may vary and may not always accurately reflect the body temperature.

The implementation of Machine Learning (ML) algorithms has had a significant impact on the healthcare industry. By using these algorithms, disease prediction methods have become much stronger, leading to the better diagnosis and treatment of patients and even saving lives [21,22]. This has made a significant contribution to healthcare and has demonstrated the effectiveness of ML in this field [23]. ML can establish the relationship between two variables by identifying specific attributes in the data and measuring the strength of this relationship using mutual information [24].

In this way, this work aims to develop a WD to monitor vital signs that integrate IoT technologies, Statistical Analysis (SA) methods, and ML models to best estimate the body temperature based on the data measured on the wrist. The contributions of this paper can be summarized as follows:

- The development of a WD for remotely monitoring vital signs, focusing on temperature measurement at the wrist and forehead. This opens the door to a less invasive and more accessible method of monitoring and measuring body temperature changes.
- The integration of the OBNiSE IoT architecture, that can be applied in general to several areas such as industry, education, mobility, smart cities, agriculture, health, energy, etc. This IoT architecture encompasses six layers: devices, network, processing, cloud, security, and applications.
- A comparative analysis of models, applying the SA and ML method, with the aim of estimating core body temperature based on wrist temperature.

This paper is organized as follows: Section 2 comprehensively reviews the related works. Section 3 shows the study case. Section 4 describes the design of the WD, including the architecture of the proposed system. Section 5 indicates the process for collecting data. Section 6 presents the applied SA and an ML algorithm to predict the forehead temperature from the wrist. Section 7 presents the experimental results and their respective analysis, while Section 8 presents the discussion. Finally, Section 9 presents the conclusions and limitations found.

## 2. Related Works

During the pandemic, rapid fever screening has proven to be an effective method for the detecting suspected symptoms of infectious diseases. However, contactless infrared thermometers are only reliable if they are properly handled.

The authors in [25] proposed the development of an infrared thermometer on the wall for fever screening. The difference with normal contactless infrared thermometers is that the prototype can automatically estimate the human body temperature when the distance is adequate to measure the forehead temperature. The results indicate that the prototype could accurately detect body temperature under various environmental and physical conditions.

The study in [26] proposed a WD to detect potential heat stroke by measuring physiological data using fuzzy logic with the wearable temperature sensor placed on the wrist. This study employs other sensors to measure the heart rate, ambient temperature, relative humidity, and core body temperature to generate a quantitative heat stroke risk level. The device can detect potential heat stroke and alert users earlier. The authors in [27] introduced an IoT-based wearable device that can help monitor vital signs related to COVID-19. The device can detect the primary symptoms of COVID-19, such as body temperature. This innovative device is designed to improve the communication between doctors, authorities, and family members, and facilitate the management of the pandemic.

The investigation in [28] aimed to test a wearable wrist device to measure vital signs. The device’s measurements were compared to the standard manual measurements of nurses, with 132 participants participating in the study. The results showed that the heart rate and systolic blood pressure measurements had high agreement with the device, but respiratory rate, temperature, and oxygen saturation measurements had low agreement. Nonetheless, most participants had a positive impression of the device and would be willing to use it in future hospital visits.

In the works presented in [29,30,31,32,33], the continuous monitoring of vital signs such as breathing and heartbeat temperatures is carried out; these variables play an essential role in predicting diseases. The results highlight the significance of assessing these devices for telemonitoring and clinical decision making, illustrating the potential of wearable technology in real-time health monitoring.

In [34], the authors propose a prototype non-contact temperature sensor for early fever detection. The thermal infrared sensor is placed on a hat to measure forehead temperature and employs a microcontroller. The data are sent to a mobile application through Bluetooth, and the estimated temperature can be visualized as a matrix. The study found that the portable wearable device can accurately detect objects with high temperatures (>37.5 °C) indoors and outdoors, with a detection accuracy of up to 99.42% and 98.10%, respectively.

Other works [35] present the development of a wearable device placed on the ear to detect early signs of COVID-19 by measuring the human biometric signals of temperature, heart rate, and SpO2. The study results reveal an average difference of 0.40 in temperature estimation compared to standardized commercial devices.

Research in [36] contemplates the best places to measure anatomical temperature using wearable sensors to measure this variable. These studies conclude that the most accurate areas to measure temperature are the forehead and the ear because they are more closely related to body temperature.

The authors in [37] propose a sensor-based wireless health monitoring system to estimate the body core temperature from the skin surface of the back under the neck. In this way, a linear regression algorithm is employed to estimate the body core temperature. The results showed that the proposed device could accurately measure skin with an average error of approximately ±0.19 °C between the ear temperature and estimated core body temperature. However, greater variability was observed in pulse oximetry measurement.

In the papers presented in [14,38], a comparison is made between temperature measurements taken in different parts of the body with infrared sensors installed on the forehead, ear, and wrist, showing the relationship between each zone; it is concluded that the temperatures taken on the forehead and ear are closest to the body temperature and that the temperature taken on the wrist usually varies, which is why it is necessary to establish a relationship with the body temperature.

Tools like ML have great potential to process large amounts of data collected by sensors from IoT devices in real environments [39]. The work presented in [40] proposes the integration of neural networks as an architecture for processing data obtained to predict diseases and abnormalities in the human body, taking advantage of these architectures’ great data processing capacity. On the other hand, the study [41] examines the potential of WD to forecast clinical laboratory outcomes through the continuous monitoring of vital signs compared to clinical vital signs. The study employs statistical and ML models, such as linear regression, Lasso, random forests, and canonical correlation analysis, to establish the correlation between measurements and laboratory results. This study demonstrates that WD can accurately predict certain laboratory tests.

In [42], WD and ML algorithms were used to continuously monitor high-risk postoperative patients and predict their outcomes in real time. The vital signs of 292 patients, including systolic and diastolic blood pressure, heart rate, pulse rate, respiratory rate, and oxygen saturation were monitored. Different ML models were trained and tested, and the results showed that the random forest model performed the best. The investigation in [43] presents an IoT system to enhance healthcare services and help professionals reduce their workload. The data were analyzed using ML techniques to detect adverse health conditions early.

The review of related works demonstrates the possibility of using IoT technologies to implement sensors and collect data remotely. It also shows the potential of implementing ML architectures to predict abnormalities in vital signs, which can help improve healthcare. This highlights the importance of ongoing research and the development of remote health status monitoring methods.

## 3. Study Case

**Figure 1:**
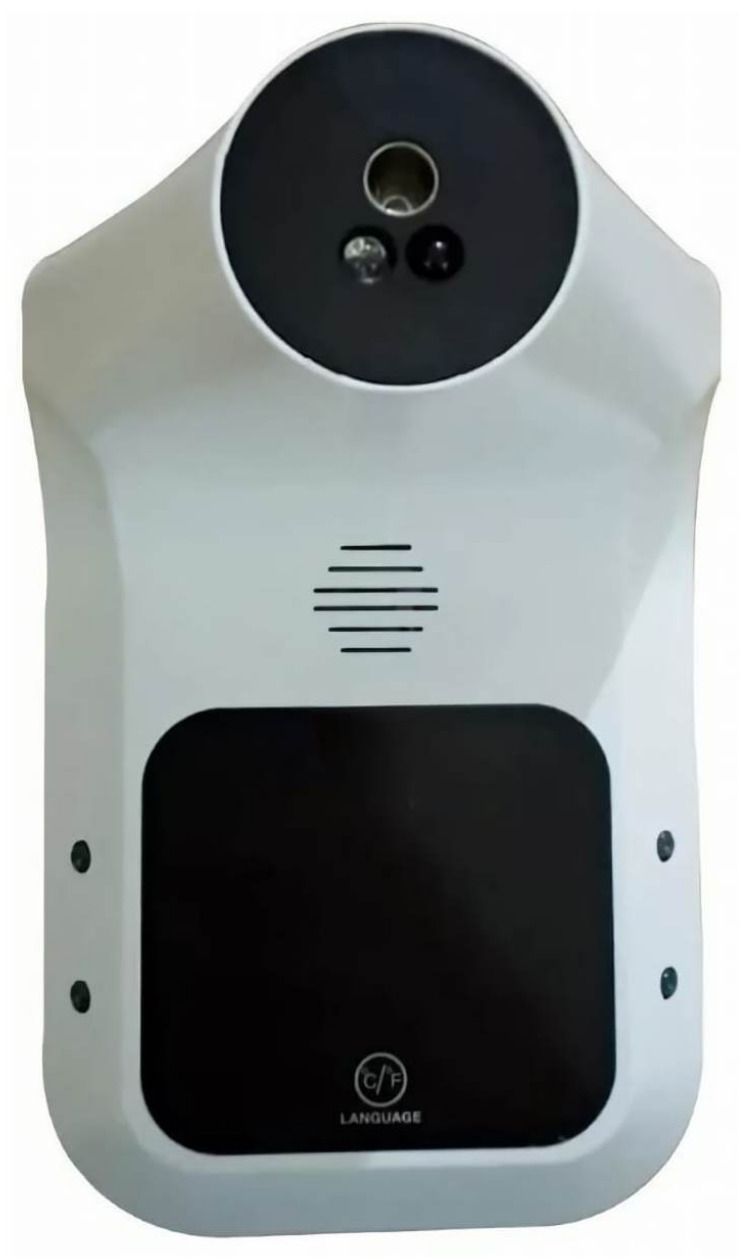
Commercial infrared sensor Model K3TIA K3 GP100 GP-100.

This study employs a commonly used temperature device used at temperature checkpoints in various locations across Mexico, namely the K3 GP-100 non-contact temperature sensor (Figure 1). The primary objective is to determine a correlation between the forehead and wrist temperature readings by obtaining the measurements of both points. The main characteristics of this device are shown in Table 1.

**Figure 2:**
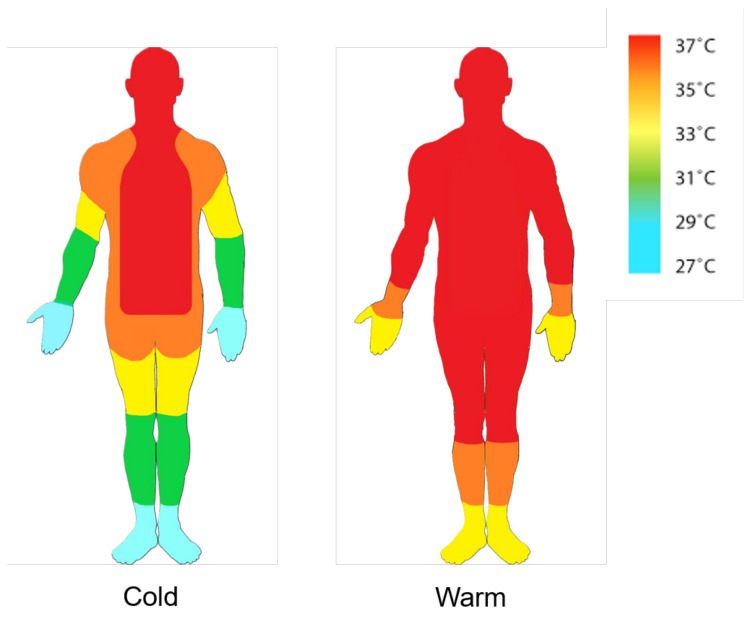
Body temperature distribution.

The experiment involved taking measurements using a mercury thermometer placed under the armpit as a ground truth under constant artificial lighting conditions. The non-contact temperature sensor is maintained perpendicular to the forehead and wrist while moving at 3 cm, 5 cm, and 10 cm for each measurement. Each measurement was sampled every five minutes, and 90 readings were taken. Research suggests that the wrist tends to have lower temperatures than other body parts, such as the forehead. The graph in Figure 2 shows that temperatures at the wrist ranged from about 31 °C to 33 °C [40].

**Figure 3:**
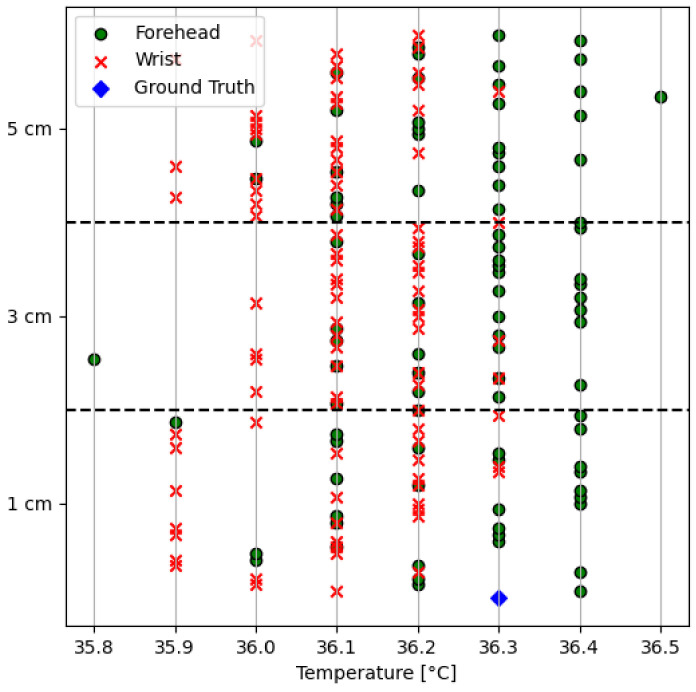
Temperature vs. distance data (reported on the x axis in °C) collected during the experiment.

Figure 3 provides the values obtained by analyzing the body temperatures at the wrist and forehead, respectively (reported in °C).

The Standard Deviation (STD) values for the forehead and wrist are 0.135 °C and 0.137 °C, respectively. Furthermore, based on ground truth measurements, the mean absolute errors for the forehead and wrist are found to be 0.107 °C and 0.196 °C, respectively. The experiment results indicate that the sensor deployed for temperature measurement does not differentiate between the temperature readings captured from the forehead and wrist. This may happen due to different factors; previous works evaluated four non-contact temperature sensors and found high bias in the measurements due to the incorrect working distance, inclination angle, measurement site, and light conditions, which can result in considerable discrepancies in body temperature estimation [44].

Determining a correlation between the temperature readings obtained from the forehead and wrist based on the provided data appears to be challenging. Therefore, to achieve accurate human body detection with minimal external impact, it is necessary to use a simple and convenient method [20].

## 4. Design and Implementation of Wearable Device

This section outlines the development of a WD for collecting body temperature using the National Digital Observatory of Smart Environments (OBNiSE, by its initials in Spanish), presented in [45], part of the Center for Research, Innovation, and Technological Development of the University of the Valley of Mexico (CIIDETEC-UVM). The OBNiSE architecture has been designed as an integrated system that enables the control, analysis, monitoring, and development of intelligent systems for the creation of IoT-based solutions while ensuring the security and efficient management of large volumes of data at various levels.

The OBNiSE architecture comprises six layers, each performing specific tasks as follows:

- Device connections: The physical layer collects all the data from the sensors.
- Network: The network comprises three essential components, namely tools, user profiles, and data accessibility. Tools constitute graphics cards, memory, and other auxiliary connections that enable the configuration of devices. User profiles are classified into three categories: programmers, managers or administrators, and regular users. Data accessibility determines how devices communicate with users and other devices, whether through Wi-Fi, Bluetooth, or ZigBee.
- Processing: This layer processes information from the network and device layers, organizes data for interpretation, and can use a physical or virtual server.
- Cloud: This layer is where the data are stored and ensures its availability for any application or service.
- Applications: This layer allows the development of services, functions, and software solutions to interact with end users created under this IoT architecture; some applications are mobility, cities, health, and technology, among others.
- Security: The system ensures control and security across all layers of architecture. Devices are stored in secure locations, user profiles are limited to authorized information, and applications use encryption. Data are stored on physical servers and the cloud, with access restricted to administrators. Encryption keys protect the information transmitted between devices, the cloud, and the server.

**Figure 4:**
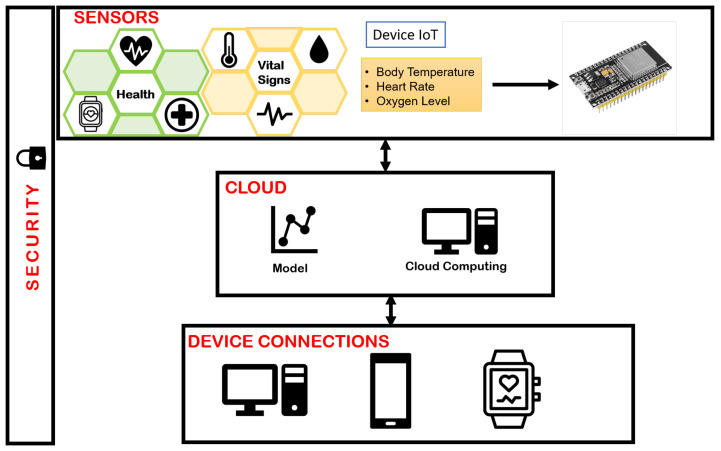
Smart system for healthcare based on the OBNiSE architecture.

The OBNiSE architecture for WD is shown in Figure 4.

The description for every layer is described in subsequent sections.

### 4.1. Device and Network Layer: Wearable Device

**Figure 5:**
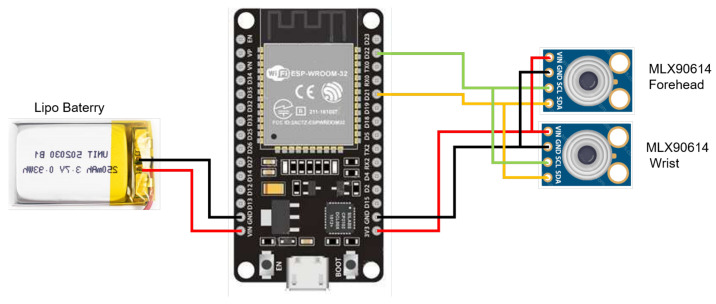
Sensors and battery connections on wearable device.

The WD consists of a microcontroller and two non-contact temperature sensors; one sensor will be worn on the wrist, while the second temperature sensor measures the forehead to collect simultaneous data between both. The connection diagram is shown in Figure 5; the elements of WD are described below.

- NodeMCU: module for managing communication tools such as Wi-Fi and Bluetooth. It can handle various applications, from low-power sensors to high-processing capacity tasks. This module features the ESP32-D0WDQ6 chip, which can be customized to meet specific requirements. It incorporates two CPU cores that can be adjusted from 80 to 240 MHz and a co-processor that conserves energy by handling simple tasks like peripheral control. Additionally, the module offers connectivity options with various peripherals, such as touch Secure Digital (SD) cards, Ethernet, Serial Peripheral Interface (SPI), Universal Asynchronous Receiver-Transmitter (UART), and Inter-Integrated Circuit (I2C).
- MLX90614: A non-contact infrared temperature sensor that integrates a low-noise amplifier, a high-resolution 17-bit ADC, and a DSP MLX90302. The calculated object and ambient temperatures are available with a resolution of 0.01 °C. They are accessible by a two-wire serial SMBus compatible protocol (0.02 °C resolution) or via the device’s 10-bit PWM (Pulse Width Modulated) output. The MLX90614 is factory-calibrated in a wide temperature ranges: from −40 to 125 °C for the ambient temperature, and from −70 to 382.2 °C for the object temperature.

**Figure 6:**
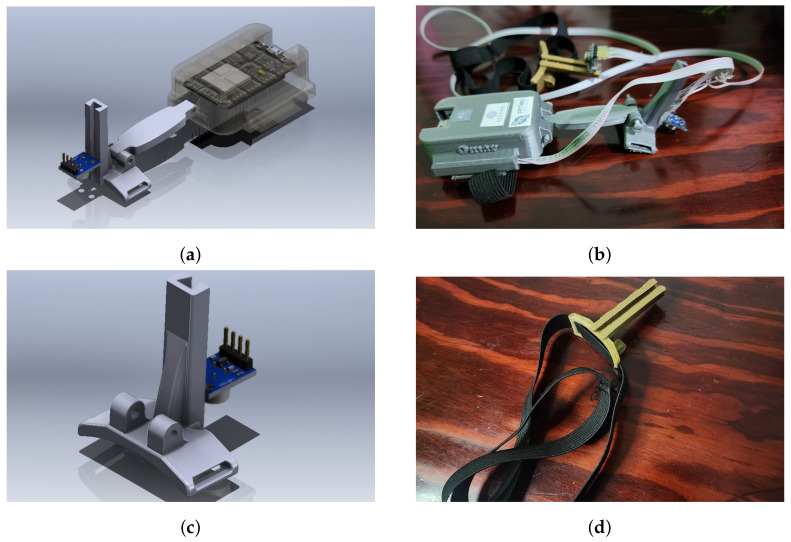
Wearable device: 3D design (**a**,**c**) and assembled (**b**,**d**).

Two enclosures were created to enable the sensors to be worn on the wrist or forehead. The enclosures possess a mechanism to regulate the distance of the sensor with the object being measured. Figure 6a,c show the wrist device, while Figure 6c,d show the forehead device.

The MLX90614 sensors and microcontroller NodeMCU are incorporated by employing the I2C communication protocol bus, with information being transmitted to the cloud via Wi-Fi using the MQTT (Message Queuing Telemetry Transport) protocol. This study establishes two types of user profiles: viewers and managers. Viewers can only view the information using a PC or mobile device, while managers can view the data, as well as modify the data processing.

### 4.2. Processing and Cloud Layer

The information is stored in a database based on cloud technology, which is available for later visualization and processing. This database can be accessed easily by both the application and the processing system. As a result, data analytics and ML techniques can be applied to the data to systematically detect trends and correlations between the forehead and wrist temperature, as explained in Section 6.

### 4.3. Application Layer

ThingSpeak is a cloud platform specifically designed for IoT and Industry 4.0 projects. It simplifies the management of device sensors, transmission channels, Message Queuing Telemetry Transport (MQTT) connections, and graphs for data analysis and storage [46]. The purpose of this study is to use ThingSpeak to present information to end-users.

**Figure 7:**
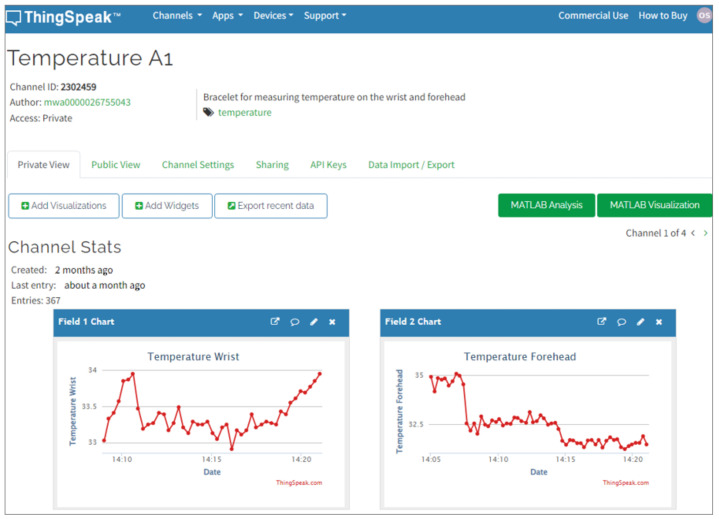
ThingSpeak cloud platform graphical interface.

An Application Programming Interface (API) allows users to import data from the cloud into ThingSpeak. The server offers graphical displays, such as dashboards, that enable users to visualize the data’s behavior. These dashboards include useful features like meters, buttons, and images, as shown in Figure 7.

## 5. Data Acquisition

**Figure 8:**
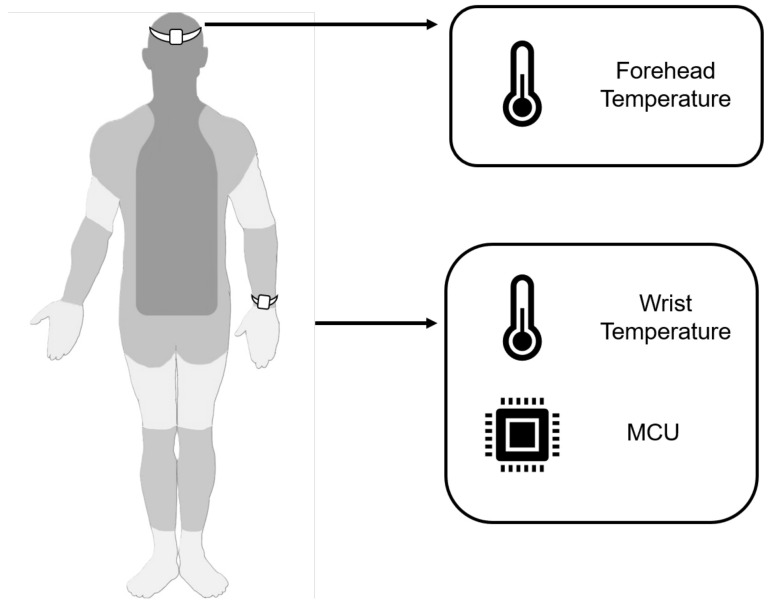
Wearable device to measure temperature in wrist and forehead.

An initial sample group of volunteers between 18 and 30 years old was called in the data acquisition process, conveniently selected by the most prevalent age of the students at the university. This group was organized volunteers aged between 19 and 24 years old and comprised 30 volunteers, 16.67% of whom were women and 83.33% of whom were men. This experiment is the first planned group. Considering the study [47] and aiming to enrich the research, the same experiment is planned for other age groups; moreover, collaborations running the same experiments, product design considerations, and ergonomy with a more ethnically diverse sample would be beneficial. Volunteers participated in an experiment to measure the temperature using sensors placed on their forehead and wrist, as shown in Figure 8. Their health data were not considered during the measurements. Furthermore, the experiment was conducted in the same room at the Autonomous University of Zacatecas under artificial light conditions and at ambient temperature.

Participants were instructed to wait for ten minutes before starting the measurement process. This allowed them to acclimate to the ambient temperature and minimize the effects of temperature fluctuations on the experiments. This is particularly important for experiments that involve a lengthy measurement process. Keeping volunteers at the same room temperature makes the measurements more consistent and reproducible, which is essential for ensuring the study’s validity. Following this, the WD sensors were attached to the participants for 15 min, with a sampling rate of 12–13 s due to IoT latency, resulting in 2130 samples.

The protocol for making the measurements is as follows. Initially, the WD sensors were employed to measure forehead temperature, placed at a distance of 1 cm, and then the distance gradually increased to 2.5 and 5 cm for five-minute intervals. Simultaneously, measurements of the temperature on the wrist were recorded by the WD at a fixed distance.

The data from 2130 samples were stored on the cloud platform to establish a correlation between the temperatures measured on the wrist and the forehead.

## 6. Statistical Analysis and Machine Learning

**Figure 9:**
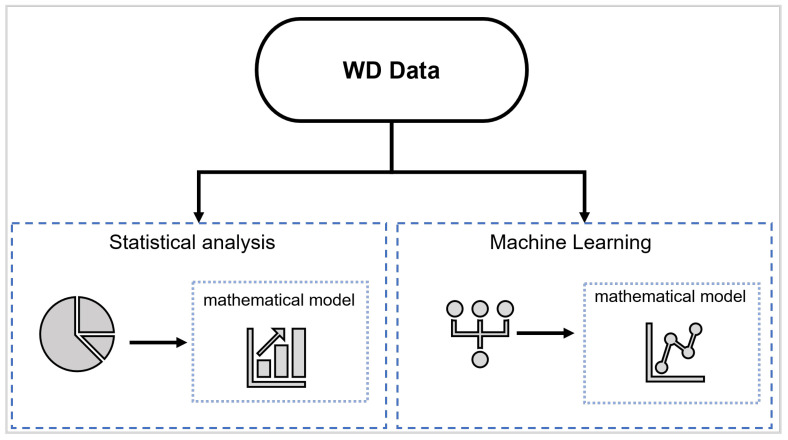
Overview of the data analysis process for obtaining the mathematical model from the wearable data.

A comprehensive analysis of the collected data will be conducted in the upcoming sections, as shown in Figure 9.

Initially, the interdependencies will be examined by generating the mathematical correlation equations for each dataset, ranging from the first degree to the third degree to capture complex patterns in the data. This approach aims to attain a detailed understanding of the relationships existing between the variables. Subsequently, ML techniques will be employed to construct predictive models. The validation of all relationships will be conducted using the coefficient of determination, denoted by R-squared (R^2^), which measures the extent to which independent variables determine the dependent variable in terms of variance proportion [48], as expressed in Equation (1):

(1) $$R^{2}=1-\frac{\mathrm{RSS}}{\mathrm{TSS}}=1-\frac{\sum {\left(y_{i}-{\hat{y}}_{1}\right)}^{2}}{\sum {\left(y_{i}-{\bar{y}}_{1}\right)}^{2}}$$

where *RSS* is the Residual Sum of Squares and *TSS* is the Total Sum.

This study involves deriving equations using SA and ML methods at three polynomial degrees. It then moves on to a crucial verification phase, where it tests the predictive efficacy of these equations using an entirely new dataset. This dataset is distinct from those used in the initial analysis, allowing the evaluation of the models’ real-world applicability and accuracy. By doing this, the study ensures that the relationships captured by the models are not just artifacts of the original datasets but hold general predictive power. The effectiveness of each equation will be assessed based on its performance on these new data, focusing on how closely the predicted values align with actual measurements. This validation step is essential for establishing the reliability and robustness of the predictive models, providing a comprehensive measure of their utility in practical applications.

### 6.1. Statistical Analysis

The SA uses a Partial Least Squares (PLS) process to obtain first-, second-, and third-degree equations. Least squares path modeling is a widely used method to analyze data associated with complex phenomena. The characteristics of PLS are essential in explaining the causal relationships between concepts in the real world. This method aims to optimally fit the polynomial equations to the data by minimizing the sum of the squares of the differences between the measured and predicted values [49].

The PLS model can be expressed as Equation (2):

(2) $$Y=B_{0}+B_{1}X+B_{2}X^{2}+\ldots +B_{k}X^{k}+E$$

where *Y* represents the dependent or response variable, *X* represents the independent predictor variable, and *k* indicates the polynomial degree. The PLS procedure estimates the model coefficients, which include $B_{0}$ (the intercept) and $B_{1}$, …, and $B_{k}$ (the coefficients of successive powers of *X*). The error term, represented by *E*, captures the discrepancy between the observed values of Y and the values predicted by the polynomial model.

### 6.2. Machine Learning

One of the most used techniques for data analysis is Linear Regression (LR) because of its ability to represent complex phenomena and ease of data understanding. The structure of simple Linear Regression is expressed as Equation (3):

(3) $$Y=\beta_{0}+\beta_{1}X+\epsilon$$

where *Y* is called the dependent variable or the response variable, whereas *X* is the independent variable used to predict. The regression coefficients or parameters of the model are represented by β0 and β1, which correspond to the intercept and slope, respectively. Lastly, ϵ represents the error in predicting the response variable due to the stochastic relationship between *Y* and *X* [48].

Polynomial regression is a type of multiple regression that only involves one independent variable, referred to as *X*. In this model, the dependent variable *Y* is linearly dependent on the powers of this single independent variable, including *X*, $X^{2}$, and so on, up to $X^{k}$. A polynomial regression model with a single independent variable and an order of *k* can be expressed as Equation (4):

(4) $$Y=\beta_{0}+\beta_{1}X+\beta_{2}X^{2}+\ldots +\beta_{k}X^{k}+\epsilon$$

where *Y* is the measured or observed variable at time *X*, the polynomial order is denoted by *k*, and the *B* denotes the parameters that need to be estimated as they are unknown. The error term ϵ is also time-dependent and follows the probability distribution of *Y*. It is important to note that t is a time sequence, where *X* equals 1, 2, 3, and so on up to *n* [50].

LR and PLS formulas are often used interchangeably because least squares is the standard method for estimating linear and polynomial regression coefficients. These equations define the relationship between dependent and independent variables, optimized using least squares. However, this study implements LR using ML techniques, making a significant difference. ML trains the model with known response data, allowing it to learn the relationship between variables and prepare to make accurate predictions on new data. This predictive capability is essential for practical applications, highlighting the importance of ML in extending SA towards broader predictive applications.

#### 6.2.1. Model Evaluation

Several theoretical aspects suggest potential connections between dependent or independent data when utilizing an algorithm to determine uninterrupted values. Selecting the appropriate metric to evaluate the model is crucial, as it aids in comprehending the phenomenon’s connection and fundamental objective. In the present study, various metrics, such as Root Mean Square Error (RMSE), Mean Absolute Error (MAE), and Mean Absolute Percentage Error (MAPE) [48] are used as follows:

(5) $$\mathrm{RMSE}=\sqrt{\frac{1}{n}\sum_{i=1}^{n}{\left(y_{i}-{\hat{y}}_{1}\right)}^{2}}$$

(6) $$\mathrm{MAE}=\frac{1}{n}\;\sum_{i=1}^{n}|y_{i}-{\hat{y}}_{1}|$$

(7) $$\mathrm{MAPE}=\frac{1}{n}\sum_{i=1}^{n}\frac{|y_{i}-{\hat{y}}_{1}|}{y_{i}}$$

where *n* is the number of observations and $y_{i}-x_{i}$ is the error between the forecast and actual values. The RMSE is a mathematical formula used to standardize the units of measurement of the Mean Squared Error (MSE). The MSE evaluates the variance by measuring how well a model fits the training data. The RMSE assigns more weight to specific data points, which results in a more significant impact on the overall error if a prediction is incorrect, as shown in Equation (5). On the other hand, MAE measures the result regarding distances from the regressor to the actual points. Unlike RMSE, MAE does not heavily penalize outliers, as it smooths out all errors due to its norm. This provides a generic and bounded performance measure for the model and can be expressed in the equation as shown in Equation (6). Finally, MAPE is used when variations impact the estimate more than the absolute values of the data, as expressed in Equation (7).

#### 6.2.2. Data Split

The dataset for the LR model was divided into 80% for training and 20% for testing. Google Colab was used as the programming editor, and Python architecture was used as the programming language to run the data modeling algorithms. The data from WD were extracted and imported into Pandas for manipulation, analysis, and use. NumPy was used for complex mathematical operations on vectors to optimize the performance. Additionally, libraries such as MatPlotLib and Seaborn were used to represent the time series graphs, and sklearn was used to design mathematical algorithms.

## 7. Results

The results section showcases the findings obtained from the phases of SA and ML, which have been outlined earlier. These findings offer a comprehensive perspective on the interdependencies assessed using mathematical correlation equations and the implementation of predictive models. The results were also validated and compared for accuracy.

**Figure 10:**
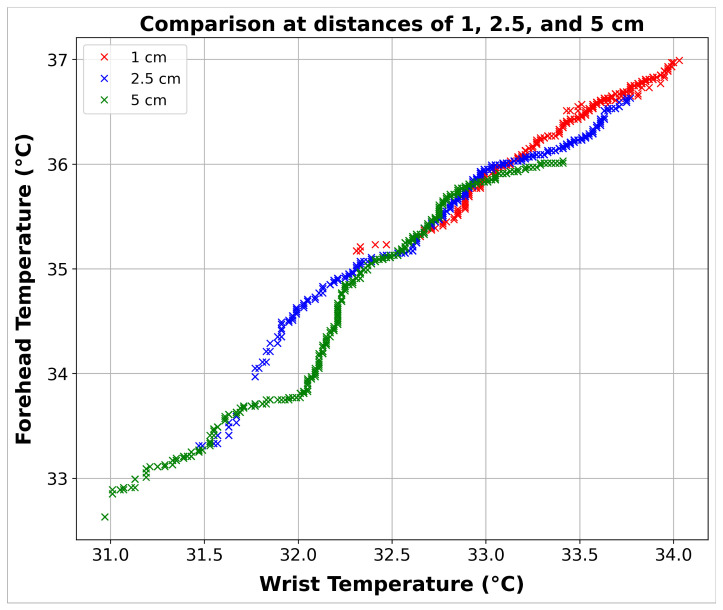
Comparison of wrist and forehead temperatures at distances of 1, 2.5, and 5 cm.

A total of 2300 data samples were gathered from 30 volunteers. These samples included wrist and forehead temperature measurements at three distances: 1, 2.5, and 5 cm. In order to conduct a comprehensive analysis, the data were systematically grouped based on distance variations. This clustering approach enabled a detailed examination of temperature readings across different proximities, ensuring the analysis was robust and granular. These grouped data are shown in Figure 10.

The data were grouped to facilitate using PLS and ML methods, which helped explore the interaction between temperature variables and develop predictive models. The categorization of data simplified the analysis process and made the findings more comprehensible and applicable in understanding the temperature dynamics among the volunteers.

### 7.1. Results for Statistical Analysis

The least squares method investigated the relationship between the wrist and the forehead temperature. Our approach focuses on implementing a correlation to identify significant patterns and quantify the strength of the association between the variables. This is achieved by obtaining correlation coefficients, which help us to determine the degree of correlation between the variables.

**Figure 11:**
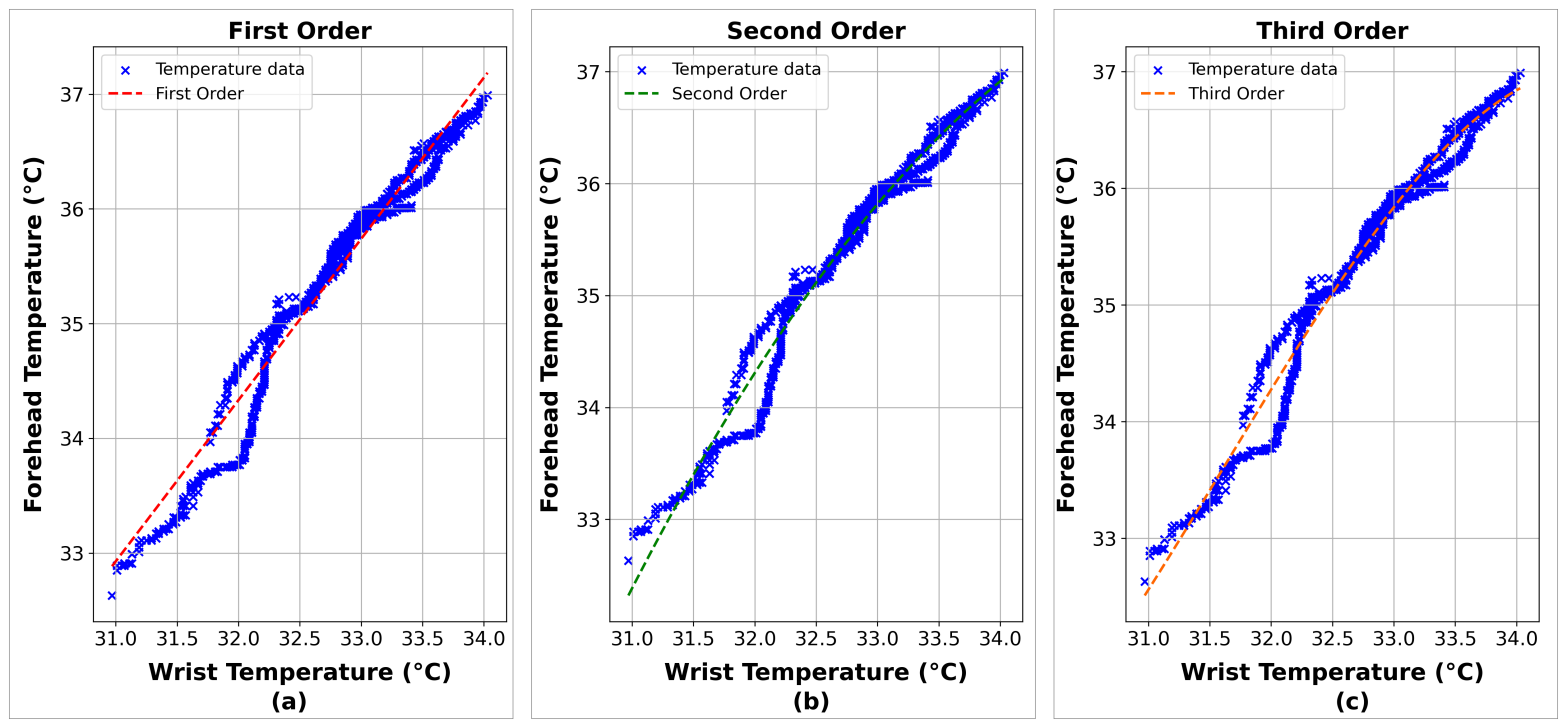
The correlation between wrist and forehead temperature using the polynomial least squares method. First (**a**), second (**b**), and third (**c**) order.

**Figure 12:**
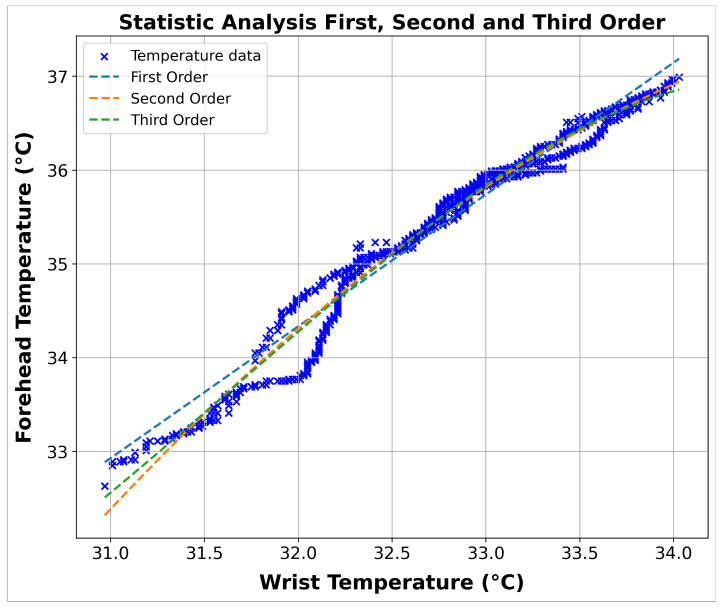
Combined visualization of the correlation between wrist and forehead temperature across first, second, and third polynomial orders using the least squares method.

The SA findings in Figure 11 and Figure 12 showcase the patterns and correlations captured by the polynomial models of varying orders, which have been fitted to the data. Table 2 provides the exact polynomial equations representing these models, which helps comprehend the modeled dynamics more clearly.

The analysis of the three polynomial models shows that they fit the data well, with all models achieving R^2^ values greater than 0.96. This indicates that the models can effectively explain the observed variability between forehead temperature and wrist temperature. The third-order model was the most accurate among the three polynomial models, achieving an R^2^ value of 0.9769. This high level of fit highlights the third-order model’s accuracy in capturing the relationship between the two variables and its superiority compared to lower-order models. This result provides strong evidence of a significant relationship between wrist and forehead temperature, emphasizing the effectiveness of the third-degree polynomial approach in modeling these dynamics.

### 7.2. Results for Machine Learning

In this study, the researcher employed ML and adopted a linear regression approach to model the relationship between wrist and forehead temperature. Forehead temperature was designated the independent variable, while wrist temperature was the dependent variable. This approach enabled an evaluation of the influence of forehead temperature on predicting wrist temperature, providing crucial insights into the interdependencies between these variables.

**Figure 13:**
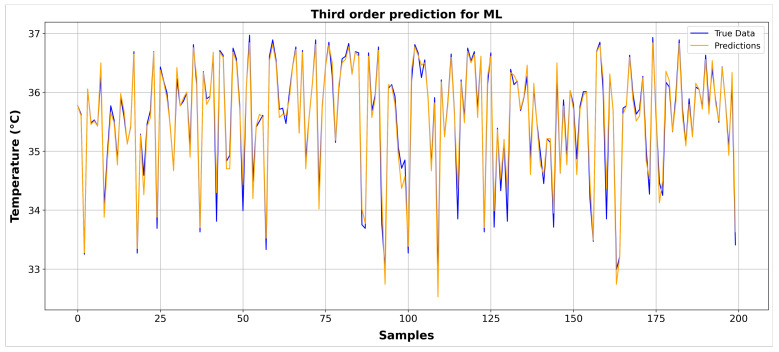
Comparison of third-degree polynomial prediction with true data for the first 200 samples.

The model was trained and tested using a split of 80% for training and 20% for testing. With 2130 samples, 1704 were allocated for training, and 426 were reserved for testing. The results of the third-degree polynomial with the first 200 samples are shown in Figure 13 compared to the true data. Additionally, Table 3 displays the training evaluation metrics for each polynomial.

**Figure 14:**
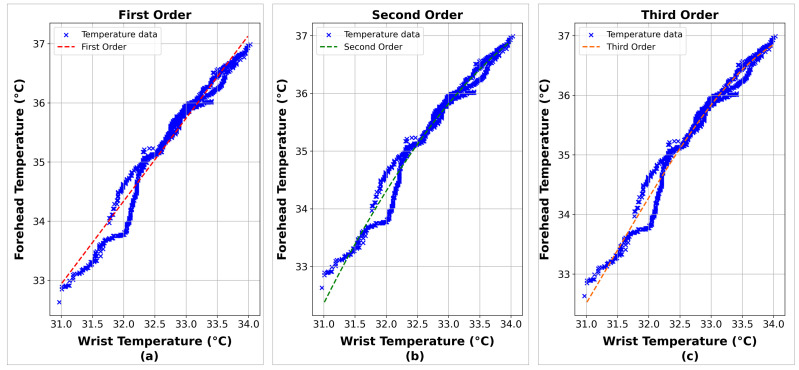
The correlation between wrist and forehead temperature using the polynomial linear regression method. First (**a**), second (**b**), and third (**c**) order.

**Figure 15:**
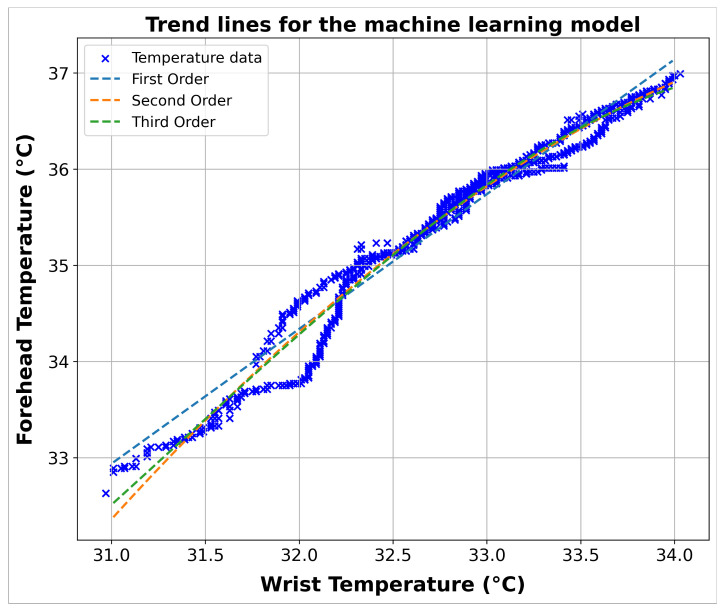
Combined visualization of the correlation between the wrist and forehead temperature across first, second, and third polynomial orders using the linear regression method.

Figure 14 and Figure 15 compare the true data and the predictions generated by the different polynomial models of various degrees, namely those of the first, second, and third order. Each graph is created to visualize the effectiveness of the corresponding model in capturing the relationship between the wrist and forehead temperature. The R^2^ values for each polynomial model are also presented in Table 4, which offers a quantitative measure of each model’s ability to explain the variation in the data. Additionally, Table 4 includes the specific equations for each model, clearly referencing the mathematical formulas underlying the predictions.

All the evaluated models demonstrated a significant fit to the data through ML, indicating the robustness of the ML techniques employed in this study. Each model effectively captured the correlation between the wrist and forehead temperature, consistent with the findings obtained through SA using least squares. However, it is important to highlight that the third-degree polynomial model was particularly noteworthy in this ML approach, achieving an R^2^ score of 0.9791.

### 7.3. Model Validation

A new dataset comprising 720 samples validated the predictive models developed for estimating the forehead temperature from the wrist temperature measurements. This dataset differed from the ones used in the model development phase and was used to test the efficacy of the six equations derived from both SA and ML approaches. The models were evaluated using two metrics: MAPE and STD. MAPE was chosen for its ability to express predictive errors as a percentage, making it easy to understand model accuracy relative to the actual temperature values. On the other hand, STD was used to measure the dispersion of prediction errors around their mean. The results of this validation process, including the predictive performance and error analysis for each model across the three polynomial degrees, are presented in Table 5.

## 8. Discussion

The validation process results, comprising 720 samples, are presented in Table 5. This dataset was used to test the efficacy of the six equations derived from the SA and ML approaches. The models were evaluated using two metrics: MAPE and STD. The results demonstrated a significant fit to the data, indicating the robustness of the techniques employed in this study. Each model effectively captured the correlation between the wrist and forehead temperatures, consistent with the findings obtained through the least squares analysis.

The results demonstrated a significant fit to the data, indicating that each model employed in this study effectively captured the correlation between wrist temperature and forehead temperature, achieving an R^2^ score of 0.9791 in the ML approach. However, the difference between the lowest and highest R^2^ shows a relatively small difference, suggesting that although the third-degree polynomial model may explain a greater variability in the data, the improvement is marginal compared to the first-degree polynomial model.

Moreover, it is important to highlight that, while the wrist temperature is not a reliable reference that reflects the body temperature [13], the data reveal that the suggested approach establishes a linear correlation between the wrist and forehead temperature. This indicates that the wrist temperature could be a viable proxy for forehead temperature, provided that the appropriate model accounts for the inherent variability in the data. Considering the model’s complexity and the risk of overfitting, balancing accuracy and complexity when selecting a model is essential. In this way, the model’s validation experiment shows a standard deviation of 1.2537 °C temperature between the wrist and forehead temperature employing the first-order ML model, achieving a difference of 0.9463 °C lower than that reported in [13].

Additionally, the fact that our data are based exclusively on temperature information may limit our study’s ability to capture the relationship between wrist temperature and forehead temperature. However, future research could benefit from including additional data, such as heart rate or skin humidity, to provide a more complete picture of this relationship. Additionally, validating our models with larger and more diverse datasets could improve our findings’ robustness and generalizability.

The wrist wearable devices currently cost around USD 60 each. However, it is important to note that this cost could significantly decrease when these devices are produced on a large scale. This price adjustment not only makes the technology more accessible for widespread use but also enhances its viability as a cost-effective solution for comprehensive health monitoring programs. In addition, the reduction in cost associated with large-scale deployments could encourage broader adoption in various sectors, including public health initiatives and remote patient monitoring systems. This would amplify the devices’ impact on human health research and care provision.

Temperature measurement in smartwatches and wristbands is an added novelty that is almost exclusive to the new models of any brand, such as Google Pixel Watch 2, Apple Watch Series 9, Samsung Galaxy Watch 6, Garmin Vivoactive 5, Huawei Watch GT-4, and others. The previous series of these devices lacked a temperature sensor. Although they are now integrating this feature, they have yet to have a clear application. This new device was created from its conception not to be another smartwatch but rather a cheap health assistant, which is why the application has been clear from the beginning.

## 9. Conclusions

The COVID-19 pandemic has emphasized the importance of remotely monitoring vital signs and efficient healthcare provision. This study introduced a WD with non-contact sensors to monitor critical health indicators, focusing on measuring body temperature as a primary health indicator. Despite the notable differences between wrist temperature and actual body temperature and considering the inaccuracy of existing devices used during the pandemic, the study explored SA and ML methods to accurately estimate body temperature from wrist temperature data. Six different models were tested, counting three using least squares-based SA and three using ML with polynomial models of degrees 1, 2, and 3. An analysis of 2130 samples from 30 volunteers showed that all models provided strong fits. However, the third-degree polynomial models stood out in both approaches, achieving R^2^ values of 0.9769 in the SA and 0.9791 in the ML. This finding underscores the superior ability of third-degree models to capture the complexity of the relationship between wrist temperature and body temperature, offering a promising solution to temperature discrepancy and the need for precision in health monitoring. The low-cost nature of the WD further enhances its appeal as an accessible tool for widespread health monitoring. This study opens new avenues for accurate and accessible body temperature monitoring, reinforcing the vital role of wearable technology and advanced analytics in the evolution of healthcare. However, further validation with larger and more diverse datasets is recommended to confirm these findings and to ensure the model’s generalizability to new data.

## Figures and Tables

**Table 1:** K3TIA K3 GP100 GP-100 sensor specifications.

Temperature range	0–50 °C
Accuracy	0.2 degrees
Response time	500 ms
Measurement distance	5 cm–10 cm

**Table 2:** Effectiveness of the polynomial least squares models for estimating forehead temperature from wrist measurements.

Model	R^2^	Equation
First-order SA	0.9634	−10.6480 + 1.4056*x*
Second-order SA	0.9761	−232.7013 + 14.9710*x*− 0.2071$x^{2}$
Third-order SA	0.9769	2120.9538 − 201.4835*x* + 6.4261$x^{2}$ − 0.0677$x^{3}$

**Table 3:** Evaluation metrics for polynomial models using linear regression.

Polynomial Oder	RMSE	MAE	MAPE
First	0.0324	0.1442	0.0041
Second	0.0218	0.0960	0.0028
Third	0.0201	0.0955	0.0027

**Table 4:** Effectiveness of polynomial linear regression models for estimating the forehead temperature from wrist measurements.

Model	R^2^	Equation
First-order ML	0.9663	−10.4919 + 1.4009*x*
Second-order ML	0.9773	−241.3410 + 15.4971*x*− 0.2151$x^{2}$
Third-order ML	0.9791	1666.7370 − 159.9030*x* + 5.1577$x^{2}$ − 0.0548$x^{3}$

**Table 5:** Detailed comparison of the validation metrics for temperature prediction models.

Model	Equation	MAPE	STD
First-order SA	−10.6480 + 1.4056*x*	0.0253	1.2554
Second-order SA	−232.7013 + 14.9710*x*− 0.2071$x^{2}$	0.0234	1.2254
Third-order SA	2120.9538 − 201.4835*x* + 6.4261$x^{2}$ − 0.0677$x^{3}$	0.0228	1.1814
First-order ML	−10.4919 + 1.4009*x*	0.0253	1.2537
Second-order ML	−241.3410 + 15.4971*x*− 0.2151$x^{2}$	0.0233	1.2254
Third-order ML	1666.7370 − 159.9030*x* + 5.1577$x^{2}$ − 0.0548$x^{3}$	0.0228	1.1878

## Data Availability

Data supporting the reported results can be found at https://github.com/Omar-Simental/Temperature_Data-UAZ- (accessed 14 February 2024).

## Abbreviations

The following abbreviations are used in this manuscript:

API	Application Programming Interface
COVID-19	Coronavirus Disease 2019
I2C	Inter-Integrated Circuit
IoT	Internet of Things
LR	Linear Regression
ML	Machine Learning
MAE	Mean Absolute Error
MAPE	Mean Absolute Percentage Error
MQTT	Message Queuing Telemetry Transport
OBNiSE	National Digital Observatory of Smart Environments
PLS	Partial Least Squares
PWM	Pulse Width Modulated
R^2^	R-squared
RSS	Residual Sum of Squares
RMSE	Root Mean Square Error
SD	Secure Digital
SARS	Severe Acute Respiratory Syndrome
SPI	Serial Peripheral Interface
TSS	Total Sum
UART	Universal Asynchronous Receiver–Transmitter
WD	Wearable Device
